# Attention allows the SNARC effect to operate on multiple number lines

**DOI:** 10.1038/s41598-018-32174-y

**Published:** 2018-09-13

**Authors:** Tina Weis, Hans-Christoph Nuerk, Thomas Lachmann

**Affiliations:** 1 Cognitive and Developmental Psychology Unit, Center for Cognitive Science, University of Kaiserslautern, Kaiserslautern, Germany; 2 Department of Psychology, University of Tuebingen, Tuebingen, Germany; 3 Leibniz-Institut für Wissensmedien IWM-KMRC, Tuebingen, Germany; 4 LEAD Graduate School and Research Network, University of Tuebingen, Tuebingen, Germany; 5 Department of Psychology, University of Leuven, Leuven, Belgium

## Abstract

To investigate whether participants can activate only one spatially oriented number line at a time or multiple number lines simultaneously, they were asked to solve a unit magnitude comparison task (unit smaller/larger than 5) and a parity judgment task (even/odd) on two-digit numbers. In both these primary tasks, decades were irrelevant. After some of the primary task trials (randomly), participants were asked to additionally solve a secondary task based on the previously presented number. In Experiment 1, they had to decide whether the two-digit number presented for the primary task was larger or smaller than 50. Thus, for the secondary task decades were relevant. In contrast, in Experiment 2, the secondary task was a color judgment task, which means decades were irrelevant. In Experiment 1, decades’ and units’ magnitudes influenced the spatial association of numbers separately. In contrast, in Experiment 2, only the units were spatially associated with magnitude. It was concluded that multiple number lines (one for units and one for decades) can be activated if attention is focused on multiple, separate magnitude attributes.

## Introduction

Despite the variety of studies investigating number processing, there is still no agreement on how (spatial) magnitudes are represented in the human brain. It is assumed that participants’ responses rely on a reference frame, an internal representation of numbers aligned horizontally in ascending order from left to right, i.e. the mental number line. Depending on the experimental paradigm, participants are able to activate either the whole number line (large reference frame) or just parts it (one or several small reference frames) that are relevant to solve the given task^1–3^. For most experiments, the numbers relevant to solve the respective task demand the activation of only one reference frame. In the present study we used two-digit numbers, for which the activation of more than one reference frame is likely to occur^3^, in order to test whether multiple spatial reference frames can be activated and operated simultaneously. To index spatial-numerical associations, we used the *Spatial Numerical Association of Response Codes (SNARC) effect*^4^.

In Western cultures (mostly using left-to-right writing) the SNARC effect is shown when participants respond faster and more accurately to numerically smaller numbers with a left key and numerically larger numbers with a right key, compared to the reversed mapping of the response keys. The SNARC effect is quite robust, and can be observed for different notations and modalities^5^ (Arabic numbers and number words; numbers presented visually or auditorily), and known to occur even when numerical magnitude is task-irrelevant^4,6,7^. The authors of the original work proposed that the SNARC effect relies on the mental number line^2,8^. According to Dehaene and colleagues^2^, the SNARC occurs for magnitude comparison (numerical magnitude is task-relevant) as well as parity judgment (numerical magnitude is task-irrelevant). Thus, not only the magnitude representation, but also its association with space in a certain direction, needs to be activated to elicit the effect. However, these two activations may not necessarily occur together. For instance, in an Eriksen task, Nuerk, Bauer *et al*.^9^ showed that, although several congruency effects indicated the magnitude activation of distractors, the SNARC effect of the distractors are non-existent or very weak. Since those spatial associations of magnitude were not activated, or were only weakly activated, the authors concluded that attention is needed to associate magnitude with spatial direction. Complementary evidence comes from a study with mathematicians^10^ who did not show a SNARC effect in a parity judgment task. However, if spatial associations are relevant for the task, mathematicians are in fact able to process magnitudes and to associate them with space^11^. Thus, it seems that the task-relevance of magnitude activation and its relation to space is important to elicit a (larger) SNARC effect.

In addition to the findings regarding the mental number line, a number of authors argue that the SNARC effect only occurs if working memory resources are available^12–14^. For example, in a study by van Dijck and Fias^15^, participants memorized a sequence of five numbers and subsequently, in a go/no-go paradigm, performed a parity judgment task on only those numbers belonging to the memorized sample. Lateralized responses were observed with the ordinal position of the numbers as presented in the sequence, but not with the magnitude of the numbers, as would have been expected in the parity judgment task. According to this study, it is not the long-term representation of numbers which results in the SNARC effect, but the creation of a task-relevant sequence in working memory. However, in a more recent study, Ginsburg and Gevers^16^ modified the paradigm proposed by van Dijck and Fias^15^. They showed that the SNARC effect and the ordinal position effect are not mutually exclusive, as was previously proposed in van Dijck and Fias^15^, but resulted from the activation of different representations: pre-existing positions in long-term memory and temporary associations in working memory^1^.

A different explanation of the SNARC effect is the theory of polarity correspondence, proposed by Proctor and Cho^17^. This general theory of compatibility effects assigns either positive or negative polarity to both the stimulus and the response. Accordingly, in a magnitude comparison task, large numbers and right-hand responses are both coded as positive polarities, and small numbers and left-hand responses are both coded as negative polarities. Given the polarity correspondence, reaction times are faster when the number and response location are SNARC compatible, i.e., they share the same polarity. Performance is worse when number and response location are SNARC incompatible, i.e., they have different polarities. However, polarity assignment can also be changed, e.g. when participants are instructed to imagine numbers in a clock-face condition^6,18^, in which larger numbers are associated on the left and smaller numbers on the right.

In line with the SNARC effect, Nuerk, Iversen, and Willmes^19^ described the MARC (linguistic markedness of response code) effect. The assumptions held by the MARC approach are comparable to those in the theory of polarity correspondence, but describe an association of linguistic markedness for the stimulus and its response. The MARC effect explains some variability in the reaction times seen in the magnitude comparison tasks and also, even more strongly, explains results in the parity judgment task. There, ‘even’ and ‘right’ are interpreted as *unmarked* conditions, whereas ‘odd’ and ‘left’ are interpreted as *marked* conditions. If linguistic markedness of both the stimulus and response are similar, i.e., the stimulus and its response are congruent, responses are faster and more accurate.

The vast majority of SNARC studies have used one-digit numbers (0 to 9). However, the effect has also been shown for multi-digit numbers^2,20–23^. The case of two-digit numbers is especially important for the present study, because they may lead to the activation of more than one reference frame. For magnitude comparison studies, it was originally argued that two-digit numbers are processed holistically on one mental number line^2^, either linearly with equally spaced steps^24^ or logarithmically^2,4,25^. However, in a series of experiments, Nuerk, Weger, and Willmes^3^ have shown that two-digit numbers are not always processed holistically, but can also be processed in a decomposed fashion^1^. Nuerk and colleagues^3,26,27^ examined the unit-decade compatibility effect. They found that in magnitude comparison tasks, compatible trials like 42_57 are processed faster and more accurately than incompatible trials like 47_62, although the distance and the logarithmic distance between those trial types were matched. In 42_57, decade and unit comparisons have the same result (4 < 5 and 2 < 7), while in the incompatible trials like 47_62, they have different, possibly interfering results (4 < 6, but 7 > 2). Thus, the unit-decade compatibility effect contradicts the interpretation of holistic processing, as it shows that decade and unit magnitudes can be processed on separate decade and unit number lines. Decomposed processing also occurs when some magnitudes are memorized and compared internally^28^.

However, and importantly, the finding that magnitudes can be processed in a decomposed fashion that may be activated simultaneously does not imply that the spatial association of magnitude is also decomposed and operating on multiple reference frames. In fact, several authors argued against the assumption of decomposed processing^21,29^. Nuerk, Bauer, *et al*.^9^ argued that the mapping of magnitude on a spatial number line requires (spatial) attention and can only occur for attended targets, but less effectively, if at all, for distractors in the Eriksen task^30^. Although the magnitudes of the distractors in the Eriksen task were clearly activated, as was shown by several effects, the spatial associations of magnitudes of these distractors were not, or only very weakly, activated for unattended distractors. Both the magnitudes of the distractors and the spatial associations of magnitudes of the distractors were strongly activated for attended targets. In sum, there is evidence for decomposed processing of magnitude. However, decomposed processing of magnitude per se *does not translate* to simultaneous *spatial*-numerical processing of magnitudes. In fact, current empirical data and theory suggest that this is not the case.

The present study aims on testing whether an experimental design can be tailored in order to activate multiple spatial reference frames in parallel. In order to build this design, two crucial manipulations were implemented. First, the spatial-numerical reference frames must be far enough apart on the number line so that single (the entire number line) and double reference frame (unit and decade number line) assumptions are clearly distinguishable. Second, the experiments were designed such that attention is not only on the units (relevant for a parity judgment task), but also on the decades, which separates the reference frames.

In two separate between-subject experiments, we focused on different aspects of number processing. In both experiments, we used the two-digit numbers 21 to 29 (leaving out 25) and 81 to 89 (leaving out 85). The spatial locations of 21 to 29 and 81 to 89 were far apart on the number line. In separate counterbalanced blocks, participants were asked to solve a magnitude comparison task by answering whether the *unit* is larger or smaller than 5, and a parity judgment task indicating whether the number is even or odd. Thus, decades are irrelevant for both these tasks. These two tasks comprise the standard trials for their respective block.

In the first experiment, between some of the standard trials, a colored fixation cross was presented, indicating a secondary task to the primary magnitude comparison or parity judgment task. These trials are subsequently termed question trials. In these cases, participants indicated whether the two-digit number presented in the preceding standard trial was larger or smaller than 50. Since the question trials were presented randomly throughout the standard trials, participants were required to focus on both the units, which were relevant for the primary task, i.e. the parity judgment or the *unit* magnitude comparison tasks, and the decades, which were relevant for the secondary task, i.e. the *decade* magnitude comparison task.

An important theoretical question was whether the focus on decades required by the secondary task (whether the number was smaller or larger than 50) is necessary to obtain multiple reference frames. Alternatively, one could suggest that an automatic activation of the magnitude of the whole number, and therefore the decade magnitude, might already be sufficient to elicit multiple spatial reference frames when they are far apart. Therefore, we ran the second experiment in which the primary task was the same as in the first experiment described above, but the secondary task asked participants to solve a color judgment task (whether the color of the fixation cross was red or green, independent from the particular number shown in the standard trial). Consequently, in Experiment 2, the two-digit number, more specifically, their decade magnitude was irrelevant – only unit parity was relevant.

Previous studies already indicated that the SNARC effect can arise even though numbers are not processed semantically, e.g. when they were superimposed on other stimuli^31^. Similar results were found in a study where attention shifts were influenced by the magnitude of a given number even though this number only served as a fixation point and thus was irrelevant to the task^32^. However, all these automatic activations refer to a single reference frame. The present study tests whether multiple spatial reference frames can be elicited without attentional focus on semantic magnitudes of decades and units, as for example in the parity judgment task the numerical magnitude was task-irrelevant and in Experiment 2 participants did not need to focus on the magnitudes of the decades at all.

**Figure 1:**
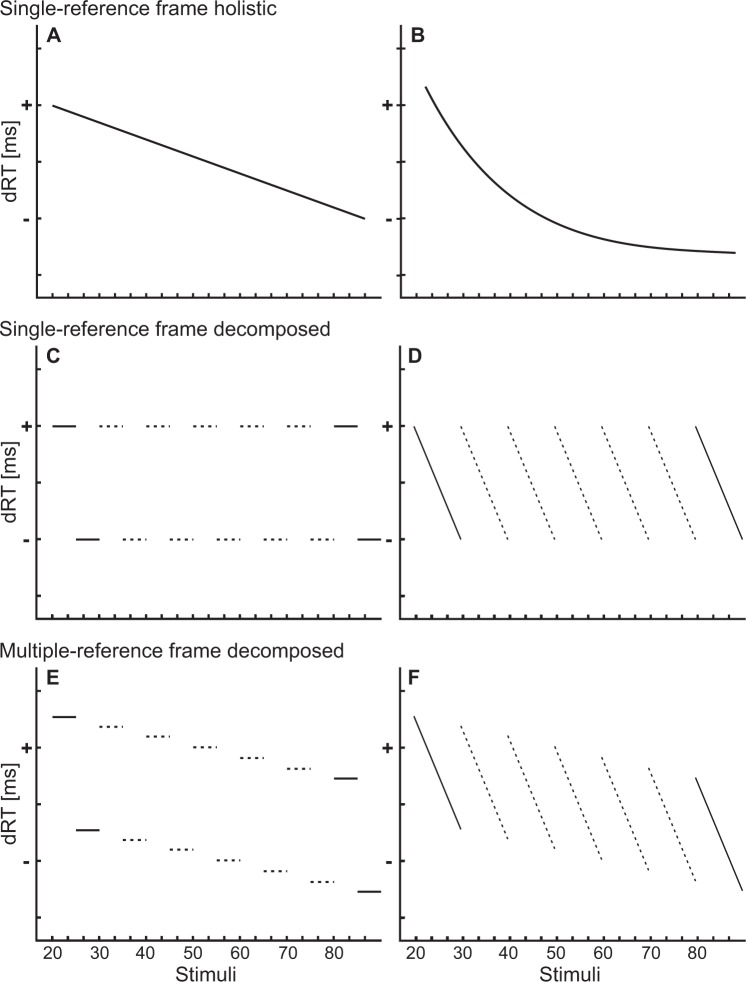
Models illustrating the different theories for two-digit number processing. (**A**,**B**) Single-reference frame holistic processing of numbers results either in a linear (**A**) or logarithmic **(B**) mental number line, because multiple-digit numbers are processed as a whole. (**C**,**D**) Single-reference frame decomposed processing of numbers, where only the task-relevant units are processed and the decades are irrelevant. This may result in a categorical (**C**) processing only indicating numbers larger or smaller than 5, or a continuous (**D**) processing with a linear decrease from 1 to 9, but no difference in the decades, which do not activate a spatial reference frame. (**E**,**F**) Multiple-reference frame decomposed processing of numbers, where both, decades and units are processed. Thus, there is an overall decrease in reaction time differences, because the decade reference frame is activated and an additional decrease within decades, because of the unit reference frame. In contrast to A and B, here 81 can be to the left of 29, because the unit 1 is smaller than 9, although 29 as a whole number is smaller than 81. Again, multiple-reference frames can be categorical (**E**) or continuous (**F**).

How can the activation of multiple spatial reference frames (decade and unit) be distinguished from the activation of one spatial reference frame for magnitude processing? To illustrate the different hypotheses for the current study, Fig. 1 presents schematic patterns of results, distinguishing holistic (single reference frame) from decomposed (multiple reference frames) results. Depicted are reaction time differences (dRTs) between left-hand and right-hand answers^33,34^, i.e., positive values indicate that stimuli are answered faster with the left hand and negative values indicate that stimuli are answered faster with the right hand.

The *single-reference frame holistic model* (Fig. 1A,B) argues for a linear mental number line and thus the processing of a digit-pair as *one* number. This model is based on the first studies about the SNARC effect^2,4,35^. Note that the holistic model can comprise of two different types, a linear coding with scalar variability^24^ (Fig. 1A) or a logarithmic coding^2,4^ (Fig. 1B). We will test for both possibilities in our study to account for the problem size effect^36^, where responses to larger numbers are slower and less accurate than to smaller numbers. If this *single-reference frame holistic model* is correct, there should be no difference between the different tasks (magnitude comparison and parity judgment) or between the different experiments (attention only to units, or attention to units *and* decades). According to both patterns of this model, numbers should be processed as a whole, independent of task requirements.

In contrast to the holistic model, a s*ingle-reference frame decomposed model* for two-digit number processing (Fig. 1C,D) assumes that only the relevant digit (units in Experiment 2) is spatially processed while the irrelevant digit is not (decades in Experiment 2). Such results would be expected when the decade does not influence spatial magnitude processing of units at all. The results for 21 to 29 and 81 to 89 would be exactly the same, because only the units were used to build a reference frame running from 1 to 9. There is only *one* spatial reference frame. However, it is not related to the magnitude of the whole number, but only to the magnitude of the units, which are relevant in the SNARC tasks. Figure 1C and 1D differ in one important aspect: Fig. 1C depicts a *categorical* SNARC effect usually found in magnitude comparison tasks^9,37^, where the magnitudes are processed in a more categorical way, indicating “larger” and “smaller” than the reference number (5, in this case). There is no obvious variation in the reaction time differences between 1 to 4 or 6 to 9, but rather there are two categories. Figure 1D depicts a *continuous* SNARC effect, usually obtained for parity judgment tasks^4,38^. The continuously decreasing reaction time differences trend line, as result of the “even” and “odd” judgment pattern intermingled within each decade, overcomes the categorical SNARC and shows a more detailed decreasing pattern. Thus, if the decades’ magnitude is task irrelevant, as in our Experiment 2, we would expect an effect only for the units as evidence for the *single-reference frame decomposed model*.

A third possibility is a *multiple-reference frame decomposed model* (Fig. 1E,F), assuming that decades and units are spatially processed in parallel, but with simultaneously occurring independent reference frames. As in Fig. 1C and 1D, the SNARC effect for the units can be seen separated for the 21 to 29 and 81 to 89, indicating that the units are clearly spatially processed. Figure 1E and 1F differ from Fig. 1C and 1D in one important aspect: In contrast to the latter, the SNARC effects in Fig. 1E and 1F are not on the same level. The SNARC effect for 21 to 29 is more towards positive dRTs, compared to the SNARC effect for 81 to 89, i.e., 21 to 29 are processed more to the left than 81 to 89. This means there is a second reference frame, indicated by a decrease in reaction time differences over decades. As such, the twenties are answered faster with the left hand, while the eighties are answered faster with the right hand. Thus, if participants had to focus on both the units and decades, as in Experiment 1, an effect for units as well as for decades would be expected on the reaction time differences, pointing towards the *multiple-reference frame decomposed model*.

Note that Fig. 1E and 1F also differ from Fig. 1A and 1B in an essential way. While, generally, both models assume that 21 to 29 are processed more left than 81 to 89, there are differences in detail. Figure 1E and 1F assume an additional unit reference frame within each decade, which partially overwrites the general left-right orientation, while in the holistic model in Fig. 1A and 1B this is not the case. In essence, the multiple-reference frame decomposed model allows that 81 is to the *left* of 29, because its unit 1 is smaller than 9, while the holistic single-reference frame does not allow this, because 81 as a holistic number is larger than 29.

These predictions allow for the derivation of the following hypotheses for the two experiments in the present study:

(i) In Experiment 1, in which the decade is relevant for the secondary task, a *multiple-reference frame decomposed model* is expected to best fit the data as displayed in Fig. 1E,F. In line with previous literature, for the magnitude comparison task, a categorical pattern is expected as displayed in Fig. 1E while a pattern displayed in Fig. 1F is expected for the parity judgment task.
(ii) In Experiment 2, where the decade is irrelevant for the secondary task, a *single-reference frame decomposed model* is expected to best fit the data as displayed in Fig. 1C,D. In line with previous literature, for the magnitude comparison task, a categorical pattern as illustrated in Fig. 1C is expected; while a trend line as displayed in Fig. 1D is expected for the parity judgment task. Alternatively, it is possible that the decade is automatically activated and thus a pattern as illustrated in Fig. 1E,F could result. However, the decade slope should then be smaller than in Experiment 1, where participants attend to decades’ magnitude.

## Experiment 1

### Methods

#### Participants

A total of 22 students of the University of Kaiserslautern (7 females, mean age = 22.9 ± 2.5 years, age range: 19–30 years) were paid for their participation in the experiment. All participants were right-handed, had normal or corrected to normal vision, and were native speakers of German. The study was approved by the Social Sciences Ethics Committee of the University of Kaiserslautern. All methods were performed in accordance with the relevant guidelines and regulations. Participants were informed about the general procedure of the experiment and were given the opportunity to ask general questions before the experiment started. Informed written consent for study participation has been obtained from all participants. However, they were not informed about the purpose of the study.

#### Task

Stimuli consisted of two-digit numbers ranging from 21 to 29 (skipping 25) and 81 to 89 (skipping 85), presented in black on a white background. The vertical visual angle of the stimuli was 2.5°.

For the magnitude comparison task, participants were asked to decide whether the unit (1 to 9) was either smaller or larger than the reference (5), irrespective of the decade. They had to answer with their left index finger on a left button for magnitudes smaller than 5 and with their right index finger on a right button for magnitudes larger than 5 in the SNARC compatible condition. Whereas, in the SNARC incompatible condition, they answered with their right index finger on a right button for magnitudes smaller than 5 and with their left index finger on a left button for magnitudes larger than 5.

For the parity judgment task, participants had to decide whether the unit (1 to 9) was either even or odd. In the MARC compatible condition, answers were given with their left index finger on a left button for odd numbers and with their right index finger on a right button for even numbers. Whereas, in the MARC incompatible condition, they answered with their right index finger on a right button for odd numbers and with their left index finger on a left button for even numbers.

At the beginning of each block, written instructions were shown along with the respective button assignment. Prior to the start of each block, there were 16 training trials, in which participants received feedback on their reaction time and correctness, displayed on the screen for 500 ms. Participants were instructed to answer as quickly and as accurately as possible.

**Figure 2:**
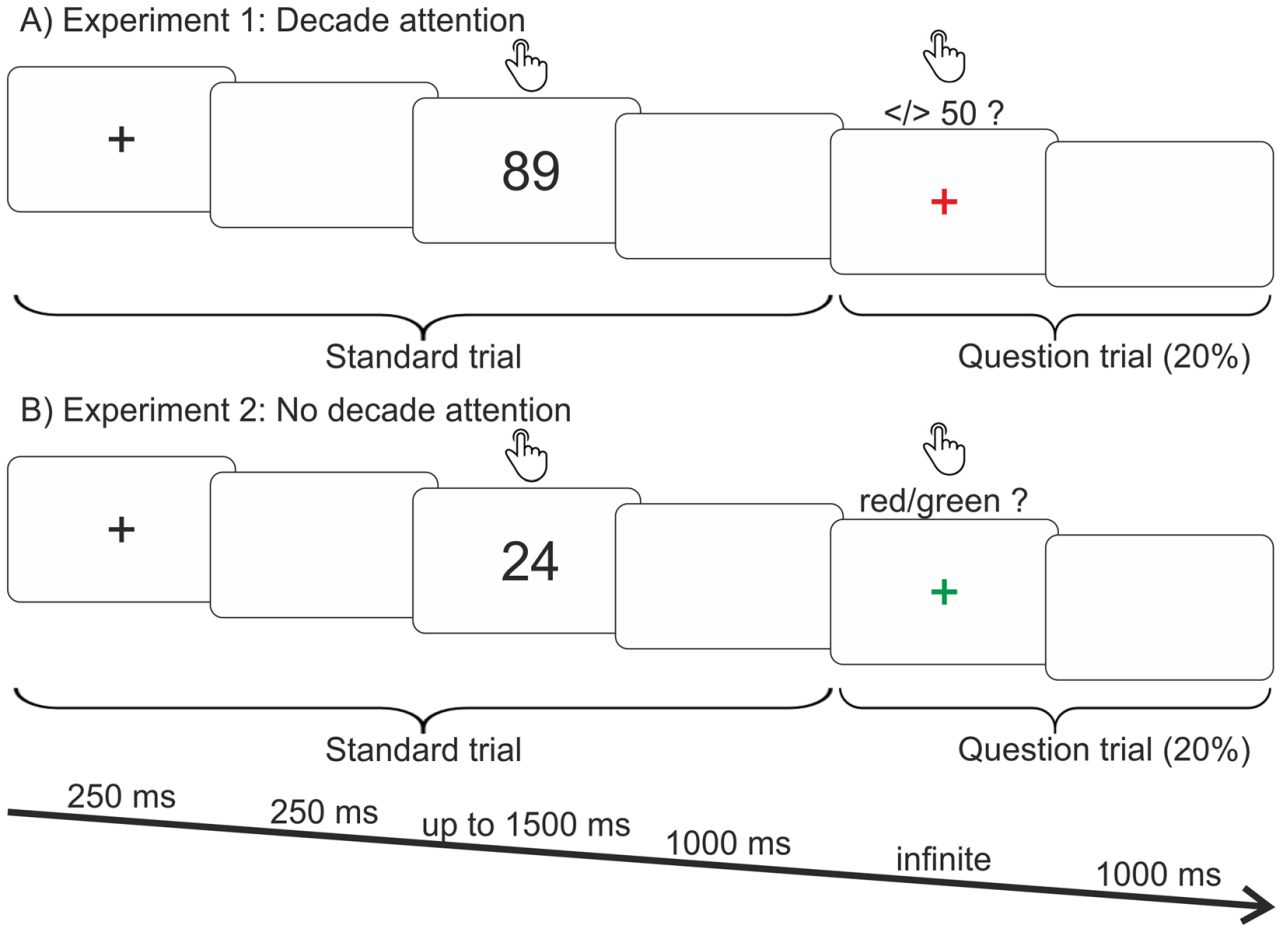
Paradigm. Participants had to solve either a magnitude comparison or a parity judgment task based on the units of the two-digit numbers presented on the screen (**A**,**B**, standard trial). In all tasks, in-between the standard trials, in 20% of the cases, a question trial was presented indicated by a colored fixation cross (**A**,**B**, question trial). In Experiment 1, participants had to answer the additional question, whether the previous number was larger or smaller than 50, after the fixation cross turned into red (**A**). In Experiment 2, participants had to answer the additional question, whether the fixation cross turned into red or green (**B**). Thus, in Experiment 1, participants had to concentrate on the two-digit number as a whole for answering the question trial, whereas in Experiment 2, the decades were irrelevant for solving the normal as well as the question trials.

Each standard trial started with a fixation cross lasting for 250 ms, followed by a 250 ms blank screen, after which the stimulus was presented for up to 1500 ms or until the participant’s response, followed by a blank screen for another 1000 ms, before the next trial begins (Fig. 2A, standard trial). In total, there were four blocks, the order of which was counterbalanced between participants: two with the magnitude comparison task (one with compatible and one with incompatible assignment of response buttons) and two with the parity judgment task (again one with compatible and one with incompatible assignment of response buttons). In each block, each of the 16 different stimuli (21 to 29 without 25 and 81 to 89 without 85) was presented ten times with random order to produce 160 trials. Over the four blocks, this totaled 640 standard trials.

Randomized within each block, in-between 20% of these standard trials, a red fixation cross was presented after participants answered the standard trial. These additional trials were termed *question* trials (32 per block, 128 in total, Fig. 2A, question trial). By instruction, this red fixation cross required participants to solve a secondary task and remained on the screen until the corresponding response was given. The secondary task referred to the number in the standard trial presented previously; participants additionally decided whether this number was either smaller or larger than 50 by pressing the left button for “smaller” and the right button for “larger”.

There was no parity judgment task for the question trials; all question trials were a magnitude comparison task. If participants focused on the number as a whole, parity judgment for the two-digit number is not meaningful since parity only refers to the units. Furthermore, instructing participants to judge the parity of the decades (ignoring the units) is not possible, as both decade numbers are even. Additionally, there was no reversed assignment of the response buttons for the question trials as there was for the response mapping of the primary task. Adding a reversed assignment to the response buttons for the secondary task may have produced interference effects in the primary magnitude comparison and parity judgment tasks. Thus, we decided to use the same question trials in all conditions to avoid confounds.

After the response to the secondary task was given, a blank screen was presented for 1000 ms, followed by the next standard trial which began with a black fixation cross (Fig. 2A).

For all standard trials, reaction times (RTs) were recorded for a maximum of 1500 ms starting from the beginning of the presentation of the number (standard trials). This is true for all standard trials, no matter if they were followed by a question trial or not, as participants were only aware of the question trial after the response to the standard trial was given. Presentation of stimuli and registration of RTs was controlled with Eprime software (Eprime 2.0, Psychology Software Tools, Inc., Sharpsburg, PA, USA) using a Hyundai Business Workstation (Windows 7, 64 bit, Intel Core i7-4790 Quad Core, 16 GB DDR4, nVidia Quadro 4100). Answers were given on a response pad (Cedrus RB-530, Cedrus Corporation, San Petro, CA, USA), using the left and right buttons. All experiments were performed in a soundproof booth with dimmed light and lasted for about 40 minutes. The stimuli were presented on a 22″ screen (LG LED Monitor) with a resolution of 1920 × 1080 pixels and 60 Hz refresh rate at maximum brightness to assure best contrast for the stimuli. Participants were placed without a chin- or head-rest at a distance of approximately 60 cm from the screen.

#### Data analysis

Reaction times shorter than 200 ms and outside ±3 standard deviations from the individual mean were excluded from analyses (3%). Data of three participants were excluded from analyses due to more than 70% errors. The data of 19 participants (5 females, mean age = 23.1 ± 2.5 years, age range: 19–30) were analyzed. There was no evidence for a speed-accuracy trade-off (correlation between RTs and error rates: r = −0.11, *p* = 0.64).

RTs for correct responses were analyzed according to the procedure described by Fias *et al*.^33,34^. In the first step of the analysis, mean RTs for each participant were calculated for each two-digit number, separately for left and right hand responses, and separated for the two tasks, magnitude comparison and parity judgment. The dRTs were calculated by subtracting the mean RT for left-hand responses from those for the right-hand responses. Consequently, a negative correlation between magnitude and dRT would be expected, because small numbers elicit faster left-hand responses (positive dRT) and large numbers elicit faster right-hand responses (negative dRT). In the second step, regression equations were calculated for each individual participant with the predictor variables *Categorical Decade* (−1 vs. +1 for 20’s vs. 80’s), *Continuous Unit* (1 to 9) and C*ategorical Unit* (<5 vs. >5) for the magnitude comparison task (see Nuerk, Bauer *et al*.^9^, for a similar procedure), and the predictor variables *Categorical Decade* (−1 vs. +1 for 20’s vs. 80’s), *Continuous Unit* (1 to 9) and *Parity* (even vs. odd) for the parity judgment task^19,39^. Note that a comparable approach by Pinhas, Tzelgov, and Ganor-Stern^40^ and Tzelgov *et al*.^39^ leads to similar results. Thus, the overall magnitude can be calculated by the following linear combination: *Magnitude* = 50 + 30 * *Categorical Decade* + *Continuous Unit*. In the third step, t-tests were performed for each predictor to test whether regression weights were significantly different from zero. These tests were performed to test not only against the null hypothesis, but also against the holistic linear model, i.e., whether the predictor *Categorical Decade* was different from 30 (as predicted by a linear holistic model). To investigate whether the resulting model was best explained by either one or two predictors, individual adjusted R² for the resulting two-predictor model was tested against the one-predictor model. To exclude any possible gender effects, additional t-tests with respect to gender were calculated. Finally, t-tests were performed on the different predictors to determine whether the slopes within the magnitude and parity judgment tasks were different. To support the results, and as an alternative to the conventional t-test, Bayes factors calculated according to Rouder, Speckman, Sun, Morey, and Inversion^41^ were also reported.

To ensure that participants solved the tasks correctly, analyses of error rates for the question trials were calculated and compared with the results of Experiment 2. However, the RTs for the question trials were not analyzed, since participants were not forced to answer as fast as possible in those trials.

### Results of Experiment 1

**Figure 3:**
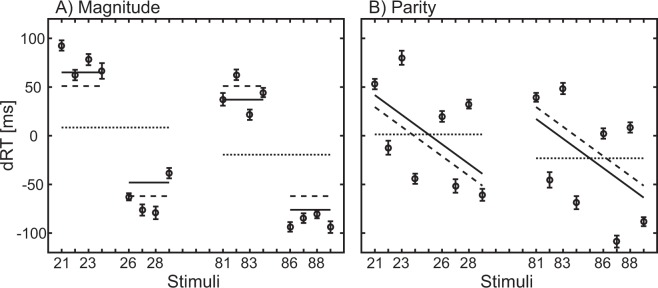
dRT results for (**A**) magnitude comparison and (**B**) parity judgment: Positive values indicate that the right-hand responses are slower: larger numbers are responded to faster with the right hand and smaller numbers with the left hand. (**A**) Magnitude comparison: dotted lines symbolize the regression equation for the significant Categorical Decade predictor only (Z = −5.53–13.99 * Categorical Decade), dashed lines symbolize the regression equation for the significant Categorical Unit predictor only (Z = −5.53–56.54 * Categorical Unit), whereas the solid lines symbolize the complete regression equation corresponding to a multiple-reference frames model: Z = −5.53 – 13.99 * Categorical Decade – 56.54 * Categorical Unit. (**B**) Parity Judgment: dotted lines symbolize the regression equation for the significant Categorical Decade predictor (Z = −10.92–12.24 * Categorical Decade) only, dashed lines symbolize the regression equation for the significant Continuous Unit predictor only (Z = −39.52–10.09 * Continuous Unit) corresponding to a multiple-reference frames model, whereas the solid lines symbolizes the complete regression equation: Z = 39.52–12.24 * Categorical Decade – 10.09 * Continuous Unit.

In the magnitude comparison task, the regression weights for *Categorical Decade, B* = −14.35*, t*(18) = −3.37, *p* = 0.003, BF_10_ = 12.83, *and Categorical Unit, B* = −66.61, *t*(18) = −3.85, *p* = 0.001, BF_10_ = 32.40, differed significantly from zero, indicating two spatial reference frames as depicted in Fig. 1E. Since there was no significant effect of *Continuous Unit, B* = −0.24, *t*(18) = −0.06, *p* = 0.951, BF_10_ = 0.24, no indication for a continuous SNARC effect was observed (see Fig. 3A). A t-test for *Categorical Decade* against 30, *B* = −14.35, *t*(18) = −10.41, *p* < 0.001, BF_10_ > 100, indicated that *Categorical Decade* influenced RTs, however, not in a manner predicted by a holistic linear model. T-tests regarding gender effects were not significant, *t(17)* = *−0.25 to 0.5, p* > *0.5*. The holistic single-reference frame model could also be rejected by testing individual R² for the two-predictor versus one-predictor hypotheses. A significant difference was obtained, *t*(18) = 4.56, *p* < 0.001, with a mean adjusted R² for the two-predictors of 0.345 compared to a mean adjusted R² for one continuous predictor of −0.01.

In the parity judgment task, the regression weights for *Categorical Decade, B* = −14.31*, t*(18) = −2.89, *p* = 0.010, BF_10_ = 5.34, and, in contrast to the magnitude comparison task, *Continuous Unit, B* = −9.28*, t*(18) = −5.52, *p* < 0.001, BF_10_ > 100, differed significantly from zero, thus supporting the multiple-reference frame decomposed model depicted in Fig. 1F. The MARC effect (*Parity)* was not significant*, B* = −1.21*, t*(18) = −0.34, *p* = 0.738, BF_10_ = 0.25. The *Parity* predictor eliminated irrelevant error variance and led to the continuous SNARC effect (see Fig. 3B). A t-test for *Categorical Decade* against 30 showed a significant effect, *B* = −14.31, *t*(18) = −8.96, *p* < 0.001, BF_10_ > 100, indicating that *Categorical Decade* influenced the RTs, however, not in a manner predicted by a holistic single-reference frame model. T-test regarding gender effects were not significant, *t(17)* = *−0.5 to 0.44, p* > *0.5*. The holistic single-reference frame model was also rejected by testing individual R² for the two-predictor versus one-predictor hypothesis. There was no significant difference, *t*(18) = 1.72, *p* = 0.102, between a mean adjusted R² for the two-predictors hypothesis of 0.065 compared to a mean adjusted R² for one continuous predictor of 0.015.

Comparison between the two tasks indicated a significant difference between magnitude comparison and parity judgment task for *Continuous Unit*, *t*(18) = 2.09, *p* = 0.050, BF_10_ = 1.41, but not for *Categorical Decade*, *t*(18) ≈ 0, BF_10_ = 0.24.

Participants solved the question trials as accurately as possible with an overall error rate of about 11%, indicating that they really processed the two-digit number.

To summarize, for both magnitude and parity tasks only the *multiple-reference frame model* could account for the data. All single-reference frame models could not account for the data, because both units and decades contributed to the spatial associations of the number, but in a way that cannot be accounted for by holistic models.

### Methods of Experiment 2

#### Participants

In total, 22 healthy students of the University of Kaiserslautern (9 females, mean age = 23.8 ± 2.6 years, age range: 20–28) were paid for their participation in the experiments. There was no overlap with Experiment 1. All participants were right-handed, had normal or corrected to normal vision and were native speakers of German. The study was approved by the Social Sciences Ethics Committee of the University of Kaiserslautern. All methods were performed in accordance with the relevant guidelines and regulations. Participants were informed about the general procedure of the experiment and were given the opportunity to ask general questions before the experiment started. Informed written consent for study participation has been obtained from all participants. However, they were not informed about the purpose of the study.

#### Task

Stimuli and overall task structure were the same as described in Experiment 1. Thus, participants solved a magnitude comparison and a parity judgment task, each in a compatible and an incompatible condition (see Fig. 2B, standard trial), but with different *question* trials (see Fig. 2B, question trial). Participants were not asked whether the preceding number was either smaller or larger than 50. Here, the fixation cross turned either green or red after the 1000 ms blank screen at the end of the standard trial. Participants were asked to decide via button press whether the fixation cross was green (right button with the right index finger) or red (left button with the left index finger). To assure comparability with Experiment 1, again, the assignment of the response buttons in the question trials throughout the different blocks was not changed. Thus, the two-digit number as a whole, more precisely the decade, did not contain any relevant information for solving the secondary task. Participants only had to concentrate on the units and could neglect the decades.

#### Data analysis

Data analysis was similar to that described in Experiment 1. RTs shorter than 200 ms and outside ± 3 standard deviations from the individual mean were excluded from analyses (2.1%). None of the participants had to be excluded from reaction time analyses due to bad performance (more than 70% errors). There was no evidence for a speed-accuracy trade-off calculated as the correlation between RTs and error rates (r = 0.19, *p* = 0.38). RTs for correct responses were analyzed as described in Experiment 1, according to the procedure described by Fias *et al*.^33,34^. To exclude any possible gender effects, additional t-tests with respect to gender were calculated.

To assure that participants solved the tasks correctly, analyses of error rates in the question trials were calculated for each individual and compared with the results of Experiment 1. RTs in the question trials were not analyzed, since participants were not forced to answer as fast as possible in those trials.

### Results of Experiment 2

**Figure 4:**
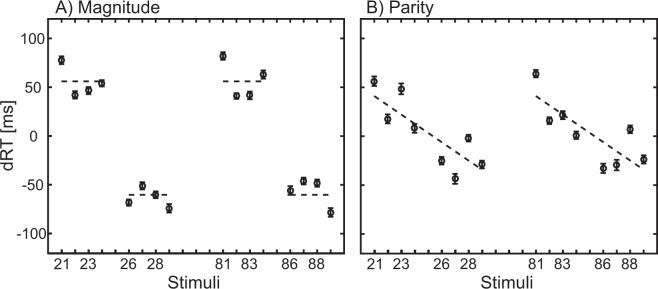
dRT results for (**A**) magnitude comparison and (**B**) parity judgment: Positive values indicate that the right-hand responses are slower; larger numbers are responded to faster with the right hand and smaller numbers with the left hand. (**A**) Magnitude comparison: dashed lines symbolize the regression equation for the significant Categorical Unit predictor (Z = −2.08–58.10 * Categorical Unit). (**B**) Parity judgment: dashed lines symbolize the regression equation for the significant Continuous Unit predictor (Z = 50.5–9.42 * Continuous Unit). Note that since the decade predictor is not significant, it is not included here like in Fig. 3.

In the magnitude comparison task, only the regression weight for *Categorical Unit*, B = −44.4, *t*(21) = −3.29, *p* = 0.003, BF_10_ = 12.25, differed significantly from zero (see Fig. 4A), whereas *Categorical Decade*, *B* = 2.0, *t*(21) = 0.47, *p* = 0.637, BF_10_ = 0.25, and *Continuous Unit*, *B* = −5.47, *t*(21) = −1.70, *p* = 0.104, BF_10_ = 0.77, were not different from zero. Hence, the data pattern reflects Fig. 1C. Although *Categorical Decade* did not differ significantly from zero, a further test for the difference from 30 was conducted and found to be significant, *B* = 2.0, *t*(21) = −6.66, *p* < 0.001, BF_10_ > 100. T-tests regarding gender effects were not significant, *t(20)* = −*1.0 to 0.6, p* > *0.5*.

In the parity judgment task, only the regression weights for *Continuous Unit, t*(21) = −4.55, *p* < 0.001, BF_10_ > 100, differed significantly from zero (Fig. 4B). *Categorical Decade*, *B* = −0.48, *t*(21) = −0.15, *p* = 0.886, BF_10_ = 0.23, and *Parity*, *B* = −4.66, *t*(21) = −1.55, *p* = 0.136, BF_10_ = 0.63, did not show any difference from zero, so that the data pattern reflects Fig. 1D. Although *Categorical Decade* did not differ significantly from zero, again a test for difference from 30 was conducted and found to significant, *B* = −0.48, *t*(21) = −9.16, *p* < 0.001, BF_10_ > 100. T-test regarding gender effects were not significant, *t(20) = −0.8 to 0.4, p > 0.5*.

Comparison between the two tasks indicated no significant difference between the magnitude comparison and the parity judgment task with regard to regression weights, *t*(21) ≈ 1.

To summarize, for both magnitude comparison and parity judgment tasks only the *decomposed-single reference frame model* could account for the data. The spatial-association of numbers was only determined by the units of the two-digit numbers, but not by the decades or the holistic value.

#### Comparison between Experiment 1 and 2

To compare both experiments, individual slopes were analyzed in a 2 × 2 × 2 ANOVA with the factors *Predictor* (Continuous Unit vs. Categorical Decade), *Task* (Magnitude vs. Parity) and *Group* (Decade Attention = Experiment 1 vs. No Decade Attention = Experiment 2). Only two of the three predictors were included since *Categorical Unit* and *Parity* are not comparable.

**Figure 5:**
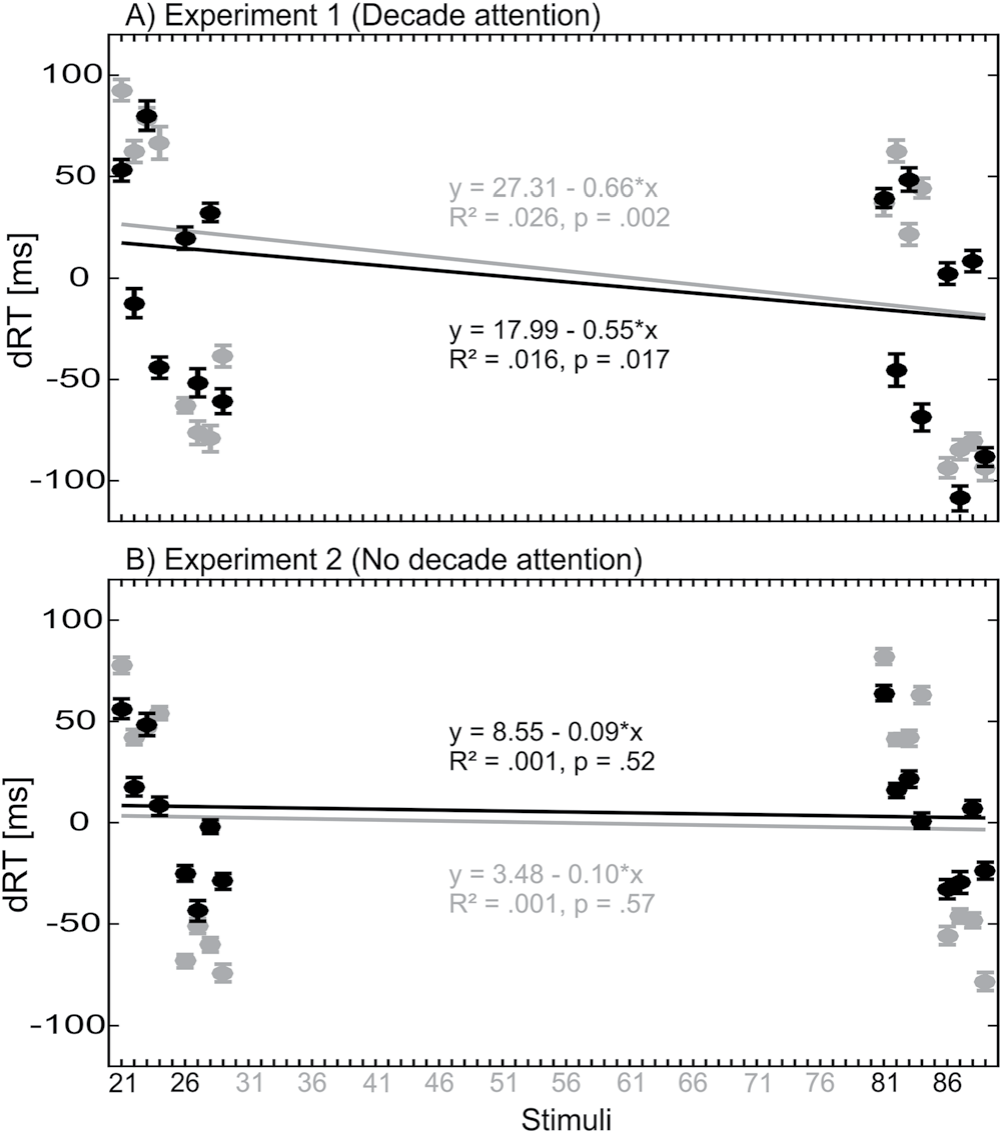
Comparison between Experiment 1 and 2 by calculating the overall slope for all stimuli (21 to 89). (**A**) Regression analyses for the slope in the magnitude comparison task (gray) and the parity judgment task (black) were significant in Experiment 1, whereas in (**B**) Experiment 2, there was no significant regression. In this graph, it can also be seen that although the decade slope differs (which is theoretically important), the main variance in spatial-numerical associations stems from the task-relevant units in all experiments.

The interaction *Predictor x Group*, *F*(1,39) = 14.1, *p* = 0.001, *η*_*p*_*²* = 0.266, indicated that there was almost no difference in individual slopes for the *Continuous Unit* (mean slope in Experiment 2, no decade attention: −7.45 vs. mean slope in Experiment 1, decade attention: −4.76), whereas there was a steeper mean slope for the *Categorical Decade* in Experiment 1 (−14.3) compared to Experiment 2, for which the slopes were not different from zero (0.76). None of the other main effects or interactions reached significance (*p* > 0.05). Figure 5 depicted the mean dRT in Experiment 1 (Fig. 5A) and Experiment 2 (Fig. 5B) for the different stimuli for the magnitude comparison task (gray) and the parity judgment task (black). Solid lines represent the overall regression for the individual tasks, including the overall regression equations. Analyses of the overall regressions in each individual task reveal a significant result in Experiment 1, for the magnitude comparison task, R² = 0.026, *p* = 0.002, slope = −0.66, as well as the parity judgment task, R² = 0.016, *p* = 0.017, slope = −0.55. There was no significant regression in Experiment 2, *p* > 0.5.

Analyses of the error rates in the question trials revealed no significant difference between the two groups, *t*(39) = 1.18, *p* = 0.24, BF_10_ = 0.53; the mean error rate in the *decade attention* group was about 11% and mean error rate in the *no decade attention* group about 9%. Thus, participants solved the question trials as accurately as possible, and the *decade attention* group really processed the two-digit number, whereas the *no decade attention* group focused on the units.

## Discussion

In line with the model predictions described in the introduction, present results indicate that multiple reference frames can be activated simultaneously in a SNARC task, even within one experiment. Thus, in the mental number line metaphor, there exist multiple spatially oriented number lines for decades and units. Their activation and access are modulated by attention, and thus dependent on the task demand. If, as in Experiment 1, both units and decades are relevant for solving the task, two different number lines are activated, because of the parallel processing of units and decades, as proposed in the *multiple-reference frame decomposed model*. Depending on whether it is a magnitude comparison or a parity judgment task, either a more categorical or more continuous number line occurs for units.

In contrast, when only the units are relevant for the task, as was the case in Experiment 2, only *one* number line is activated and accessed, representing the numerical magnitudes of the units, irrespective of the decades, as assumed by the *single-reference frame decomposed model*. Again, the effect for units is dependent on the task, with a categorical number line for the magnitude comparison task and a continuous number line for the parity judgment task. There is no effect for decades, since the task did not require attending to them.

It may thus be concluded that multiple or single number lines can be activated in a SNARC task, depending on the attentional demands of the task. When the decade can be ignored or inhibited, only a single number lines is activated. Several studies^3,42^ have shown that the magnitude of a digit in a two-digit number is activated, at least to some extent, even if it is 100% irrelevant. However, automatic activation of magnitude of a constituent digit does not mean that this magnitude is automatically mapped to space. This mapping seems to require attention, which can even be divided, such that multiple number lines are activated.

Finally, the *single-reference frame holistic model* is not able to explain the present results because there is neither a linear nor a logarithmic single number line. Essentially, any holistic model would predict 81 to be associated far to the right from 29. This is, however, not what was found in the present study. In all experiments, 81 (or other lower numbers in the eighties) was associated with a more leftward response than 29 (or other higher numbers in the twenties, see Figs 3, 4). Any purely holistic model will fail to capture this data pattern because the overall (linear or logarithmic) magnitude of all eighties numbers is much higher than the twenties.

### On the task relevance of activation of multiple reference frames

One particularly interesting effect found in the present study was that the *multiple-reference frames model* was only confirmed for data from Experiment 1, where magnitude of the decades was task-relevant, but not for data from Experiment 2, where the decades were irrelevant. How does this fit to the literature? Fischer *et al*.^32^, showed that *for single-digit numbers* (i.e., one spatial-numerical reference frame), attentional shifts to either the right or left visual field occur when presented numbers were irrelevant to solve the task. Thus, for a single-digit task, automatic activation of a single spatial reference frame is possible. However, some studies failed to replicate these findings^43,44^. In these studies, attentional effects were found only when a magnitude comparison task was involved. The authors concluded that perceiving numbers does not automatically lead to attentional shifts, which modulate a SNARC effect. Furthermore, they concluded that numbers do not follow the organization of the mental number line but the organization is response- as well as memory-related. In contrast to those studies which used one-digit numbers, Zhou *et al*.^21^ investigated the effects of serial or simultaneous presentation of the two-digit numbers. They found that numbers presented serially are processed holistically. However, this claim was later refuted by Moeller, Nuerk *et al*.^28^, who showed that the lack of decomposed presentation with an internal standard is due to stimulus attributes and choice. Even in Zhou *et al*.^21^, the compositional model explains the results for simultaneous presentation. Thus, both magnitudes can be activated automatically^3,42^, even when one digit is 100% irrelevant in the task. However, this does not imply that the spatial associations of these magnitudes are automatically activated. Zhou *et al*.^21^ found a SNARC effect for either whole numbers or decades, but not for units, and concluded that two-digit numbers were represented on one single mental number line. Thus, the multiple-reference frame was not valid for explaining their data.

How can this be reconciled with the present data? One possible explanation is that it lies in the different tasks used. Zhou *et al*.^21^ used a number matching task, while magnitude comparison and parity judgment tasks were used in this study. Nuerk *et al*.^27^ proposed a taxonomy for multi-digit number processing, in which they distinguished place-value identification, place-value activation and place-value manipulation (e.g., in carry operations in addition). Zhou *et al*.’s visual matching task was at the level of identification so no semantic processing of the numbers was necessary, as semantic processing occurs automatically and is not task-relevant. In contrast, the tasks performed in this study were semantic tasks, in which numerical attributes of the digits had to be accessed. The secondary tasks, for possible decade activation, were either semantic (Experiment 1: Magnitude comparison) or non-semantic (Experiment 2. Color judgment). Multiple reference frames appear to be activated only if both decades and units have to be semantically processed and if (divided) attention focuses on the semantic attributes of both. It seems that a simple number matching task (being at the place-value identification level) is insufficient to elicit multiple reference frames.

One could argue that the activation of the different number lines occurs either simultaneously or successively on a smaller time scale. Since both number lines interfere with each other, the activation has to be fast, either fast sequentially or even overlapping. Future experiments with different stimulus-onset asynchronies might be able to differentiate between both interpretations.

The question of holistic or decomposed spatial processing is not restricted to numerical items. Lachmann and van Leeuwen^45–48^ and others^49,50^ found a distinction in preference for analytic versus holistic processing of letters versus non-letter shapes. These studies revealed that while a holistic strategy is preferred for processing non-letter shapes, an analytic, less context-sensitive strategy is preferred for processing letters. However, the authors found that the preference of an analytic strategy for letter processing depends upon the task^47,48,51^. As such, a distinction between analytic and holistic processing strategies was only found in those tasks where a differentiation between letters and non-letter shapes was required or proved beneficial. When this was not the case, holistic processing was applied for both letters and non-letter shapes. This indicates that participants automatically elicit a strategy most appropriate to solve the task at hand, either more holistically or more analytically. In the numerical task, they select either the single-reference frame holistic or decomposed strategy, as long as it is beneficial to solve the task. When the task requirements change, however, participants are able to automatically adjust their processing strategies towards the multiple-reference frame decomposed model. Moreover, Lachmann and van Leeuwen^46^ showed that the preference for an analytic strategy in letter processing depends on the reading ability of the participants. Children with poor reading skills tend to use a more holistic strategy for both letters and non-letter shapes, whereas skilled readers tend to use a holistic strategy for non-letter shapes and an analytic strategy for letters^46^. Thus, future studies investigating the activation of different strategies for number processing should take into account participants with difficulties in number processing or children just starting to learn the association of numbers with the mental number lines.

Another factor influencing the mental number lines might be the number-word systems that differ across languages. In this study, only native German speakers participated. Spoken two-digit numbers in German start with the unit, followed by the decade (“ein-und-zwanzig”, “one-and-twenty”). Thus, the German number word system follows an inversion property. Depending on the different strategies participants might use for solving our task, it could help them to memorize the whole numbers in Experiment 1 if they were spoken aloud or even subvocally. Thus, this might explain the results where the participants show multiple number lines in the current study. This may occur because the decade is in the last position when the participant “read” the numbers and it’s kept in working memory to help them better remember the decades. On the other side, the stronger focus on the units, as they are processed first, might lead to a stronger attention for these, and thus to the reliable SNARC effect for units. Future studies should take into account differences between languages. In English or Spanish, for instance, the unit follows the decade and thus it might be harder to remember the decade. Therefore, a second number line arising for the decades might not appear. Several studies indicate that the unit-decade compatibility effect depends on language^52–56^ (but see Bahnmüller *et al*.^57^, for limits of language effects). Macizo and Herrera^52–54^ were able to show that Spanish participants can focus more easily on decades and ignore the units. The same is true for English, where the focus is also on the decades instead of the units, whereas in German the focus is on the latter^58^. Therefore, it is clear that language can influence multi-digit number processing on multiple levels (see also their special issue^27,59^). Decomposed processing can be influenced by language properties. However, as seen above, the decomposed activation of magnitudes does not automatically translate into a decomposed multiple reference frames model for spatial-numerical associations. Therefore, cross-lingual studies need to examine whether language modulation also holds for the activation of multiple number lines.

There are two indications that this might be the case. First, the SNARC effect for single-digits (i.e., for one spatial references frames) is influenced by language properties^60^. Secondly, explicit spatial processing^61^ of two-digit numbers is also influenced by language, at least in children^62^. This may suggest that implicit spatial-directional associations of numerical magnitude are also influenced by language when they operate on multiple reference frames, but this remains to be shown.

Furthermore, there is some evidence that gender differences might affect the spatial representation of numbers^63^. Bull *et al*.^63^ showed a male advantage in number-line estimation, parity judgment and magnitude comparison tasks. In line with those results, Reinert *et al*.^64^ also found a male advantage effect in number-line estimation. In contrast, in the current study, no gender-related effects were observed. Compared with the results of Bull *et al*.^63^, they found the strongest effect in the number-line estimation, whereas the effect in the magnitude comparison task was weaker. Hormonal effects are – to the best of our knowledge – usually not strongly related to numerical/arithmetic performance, even though brain activation can be affected^65^. Of course, this does not preclude differences in the more spatial-numerical tasks. However, most of the studies on the SNARC effect did not control for gender or hormonal effects at all and did not report any gender effects.

### Summary and Perspectives

The current study shows that the SNARC effect and in particular spatial-numerical associations can operate in multiple reference frames for decomposed numbers. However, this is not automatically the case, but only when the constituent numbers are task-relevant and semantically processed. Nevertheless, under such conditions, multiple spatially-oriented number lines can be accessed or activated simultaneously – spatial activation of a number is not restricted to one holistic or decomposed entity of that number.

Another important take-home message from this and previous studies is that it is necessary to dissociate the activation of magnitude and the activation of a spatial association of magnitude. Previous studies have shown that decomposed magnitudes of two-digit numbers can be activated even when one digit is task-irrelevant. This does not seem to be true for the spatial association of magnitude. Here task-relevance and attention on the task-relevant digits seem to be fundamental for the activation of multiple spatial reference frames of different magnitudes.

In essence, the spatial-numerical association of number magnitude and perhaps other stimuli, such as letters, is a complex multi-dimensional construct that seems to be modulated by task demand, attention, working memory, and language if there exists an automated processing strategy.
